# Non-participation and attrition in a longitudinal study of civilians exposed to the January 2015 terrorist attacks in Paris, France

**DOI:** 10.1186/s12874-020-00943-x

**Published:** 2020-03-14

**Authors:** Cécile Vuillermoz, Lise Eilin Stene, Lydéric Aubert, Yvon Motreff, Philippe Pirard, Thierry Baubet, Sophie Lesieur, Pierre Chauvin, Stéphanie Vandentorren

**Affiliations:** 1 Centre National de la Recherche Scientifique (CNRS), Centre Maurice Halbwachs (CNRS-UMR8097, EHESS, ENS), F75014 Paris, France; 2 Norwegian centre for violence and traumatic stress studies (NKVTS), Oslo, Norway; 3 Santé publique France, Direction des régions, F94415 Saint-Maurice, France; 4 Department of Social Epidemiology, INSERM, Sorbonne Université, Institut Pierre Louis d’Epidémiologie et de Santé Publique, F75012 Paris, France; 5 Santé publique France, Direction des maladies non transmissibles et traumatismes, F94415 Saint-Maurice, France; 6 CESP Inserm 1178, Université Paris 13, Paris, France; 7 Psychopathology Department for Children, Adolescents, General Psychiatry and Specialized Addiction, APHP Hôpital Avicenne, F93009 Bobigny, France; 8 Centre national de Ressources et de Résilience (CNRR), Paris, France

**Keywords:** Attrition, Non-participation, Bias selection, Terrorist attack survey, Mental health survey

## Abstract

**Background:**

Non-participation and attrition are rarely studied despite being important methodological issues when performing post-disaster studies. A longitudinal survey of civilians exposed to the January 2015 terrorist attacks in Paris, France, was conducted 6 (Wave 1) and 18 months (Wave 2) after the attacks. We described non-participation in Wave 1 and determined the factors associated with attrition in Wave 2.

**Methods:**

Multivariate logistic regression models were used to compare participants in both waves with those who participated in the first wave only. Analyses were performed taking the following factors into account: socio-demographic characteristics, exposure to terror, peri-traumatic reactions, psychological support, perceived social support, impact on work, social and family life, and mental health disorders. Characteristics of new participants in Wave 2 were compared with participants in both waves using a chi-square test.

**Results:**

Of the 390 persons who were eligible to participate in the survey, 190 participated in Wave 1 (participation rate: 49%). The most frequently reported reason for non-participation was to avoid being reminded of the painful event (32%, *n* = 34/105). In Wave 2, 67 were lost to follow-up, 141 people participated, of whom 123 participated in Wave 1 (re-participation rate: 65%) and 18 were new. Attrition in Wave 2 was associated with socio-demographic characteristics (age, French origin) and location during the attacks, but not with terror exposure or mental health disorders. Compared with those who participated in both waves, new participants declared less social and psychological support since the attacks.

**Conclusions:**

Attrition at 6 months was not associated with exposure to terror or mental health disorders, which indicates that any bias in future analyses on IMPACTS on mental health outcomes will be limited. Our findings suggest the importance of adapting similar surveys for people of foreign origin and of improving strategies to avoid attrition of younger people, for example by using social media, peers, and the educational environment. The present study also revealed that a high level of exposure to terror and a lack of social and psychological support after a terrorist event could impede individuals’ participation in similar surveys in the short term.

## Background

In January 2015, a series of terrorist attacks in the greater Paris area commenced with the massacre of members of the satirical magazine Charlie Hebdo by two terrorists in the city centre. A few hours later, a third terrorist injured an individual in a nearby town, and the next morning killed a police officer in another suburban town. The following day, he took hostages in a grocery store and killed 4 of them [1–3]. In total, these three terrorists killed 17 people and injured 20 people in 3 days.

In order to investigate the impact of these terrorist attacks on civilians and on rescue workers’ mental health and social functioning, as well as to assess the social support and mental health care they received in short- and in long-term care, *Santé publique France* (the French National Public Health Agency), with the support of the Greater Paris regional health agency, launched the IMPACTS survey (the French acronym for Investigation of Trauma Consequences in People Exposed to the January 2015 Terrorist Attacks and their Support and Mental care) [4]. The second wave of the survey was implemented with the collaboration of INSERM (the French National Institute of Health and Medical Research).

Despite the frequency of terrorist attacks worldwide and increased numbers in recent years in Western Europe, few epidemiological surveys have studied their health impact because of methodological and ethical issues [5]. In particular, their unpredictability makes it difficult to begin an investigation, while conducting research on potentially traumatized individuals immediately after the event requires rigorous ethical considerations and healthcare follow-up procedures.

The few studies that have been conducted on individuals involved in terrorist attacks rarely examine methodological issues like selection bias. Generally speaking, non-participants in health surveys are more likely to be male, single or divorced, with a lower educational level, unemployed, and/or with poor health [6–9]. In mental health surveys, non-participation may be associated with the type and the level of exposure and with the outcome of interest itself [10]. In particular, in studies on traumatic events, people with severe physical disabilities or injuries and people with mental health disorders are less likely to participate [11, 12]. Furthermore, disaster-related factors such as exposure to terror may influence participation. For example, people with a low exposure level may feel that their participation is less legitimate. Similarly, those with a high exposure level may prefer to avoid being reminded of the event [13].

In longitudinal surveys, attrition (loss to follow-up) may be associated with demographics and socioeconomic characteristics, health status, health-related behaviours, and healthcare experiences [14–16]. In the specific case of longitudinal trauma surveys, attrition may be higher in people whose physical and/or mental health deteriorates over time, especially in the case of intentional trauma like terrorism, war or torture [17–20].

To enhance participation and to minimize the risk of bias, previous research proposed open cohorts, where eligible persons who do not initially participate in the first wave of a study may be re-contacted and included in future waves [21]. IMPACTS was designed as an open cohort study, the initial waves being conducted 6 and 18 months after the January 2015 attacks (specifically, between June and October 2015, and between June and October 2016).

In the present study, we aimed to describe reasons why some civilians exposed to terror did not wish to participate in Wave 1 of IMPACTS, to determine the factors associated with attrition in Wave 2 in order to describe any selection bias, and to compare characteristics of new participants in Wave 2 with participants of both waves in order to examine the benefit of the open cohort design. To do this, we studied whether sociodemographic characteristics at baseline, terror exposure characteristics, peri-traumatic reactions, psychological support given by professionals, social support and certain diagnosed mental health disorders were different between 1) participants in both waves, 2) participants in Wave 1 who were subsequently lost to follow-up in Wave 2, and 3) participants not included at Wave 1 but who participated in Wave 2.

## Methods

### Design and population of IMPACTS survey

The design of the IMPACTS survey has been described elsewhere [4]. Wave 1 was conducted between June and October 2015 among four civilian sub-populations:

1. Persons listed either by the authorities, or by CUMP (Medico-Psychological Emergency Unit) volunteers as injured, a hostage or a witness who had to flee the scene because their lives were threatened (*N* = 410). They were contacted by telephone by health professionals;
2. Members of the editorial staff of the *Charlie Hebdo* magazine (*N* = 15). The IMPACTS survey was introduced to them face-to-face by the survey’s investigation team. Afterward, they were contacted by email;
3. Residents and workers within a 100-m radius of the sites of the attacks (*N* = 884). Letters were sent to 1295 households, accounting for an estimated 2635 residents. Using INSEE (the French National Institute of Statistics and Economic Studies) data, the investigation team estimated that 10% of the resident population (264/2635) were at home during the attacks. Letters were also sent to 72 companies where approximately 620 workers were present during the attacks;
4. Civilians identified by other victims through snowball sampling [22]. They were contacted by telephone by health professionals.

The inclusion criteria for IMPACTS were: being a civilian from one of the 4 categories mentioned above, aged 16 or over, and meeting one of the 4 A (i.e., stressor) criteria for Posttraumatic Stress Disorder (PTSD) as set out in the Diagnostic and Statistical Manual of Mental Disorders, Fifth edition (DSM-5) [23]. In terms of the latter, the following four exposure categories were defined:

- directly threatened: suffering from physical injuries, taken hostage, or present at the scene of the event scene and exposed to at least one of the following situations: eye contact with/heard the voice of/talked with the terrorists; seen a weapon pointed directly at them.
- indirectly threatened: both directly present at the scene during the attacks - but not in the category “directly threatened” - and having at least one of the following exposures: seen/heard someone else being threatened/being injured/dying; seen blood or inert/dead bodies; touched injured/inert/dead bodies, smelled gunpowder.
- witnesses: at home or working within a 100-m radius of the events and not in the categories “directly/indirectly threatened”
- close relatives of those who were murdered, injured and/or taken hostage.

After inclusion, in both study waves, participants were interviewed face-to-face by trained trauma psychologists. A training day was arranged for all psychologist: theoretical training, presentation of the tools, practical workshops in small groups.

Wave 2 was conducted 1 year after Wave 1 (i.e., 18 to 22 months after the events), between June and October 2016. All the participants who participated in Wave 1 were re-contacted by phone by 26 of the 31 psychologists who intervened in Wave 1. Additionally, people who responded to the inclusion questionnaire and were eligible for the study but did not participate in Wave 1 were contacted for Wave 2 by phone.

### Study variables

As mentioned above, non-participation and attrition may be associated with socio-demographic characteristics [6–9], high-level exposure to a traumatic event [10–13], and poor mental or physical health (as a direct consequence of the traumatic event in question or not). In addition to these factors, for the present study, we made the hypothesis that survey participants interviewed by media about the January 2015 terrorist attacks before either wave, may have been either less apprehensive or, alternatively, more reluctant to talk again about their experience .

#### Sociodemographic characteristics

Sociodemographic data collected in both waves included: sex, age at time of Wave 1, French origin (yes/no), educational level (higher or lower than high-school diploma), occupational status (employed/unemployed) and living with someone (yes/no) at the time of Wave 1.

#### Terror exposure and peri-traumatic reactions

Terror exposure was measured using several indicators: geographic exposure (less than 10 m from terrorist(s), very close or in the next room to the attack, in a neighbouring building or street, or elsewhere), exposure category (see above), and perceived terror exposure as measured by an analogical scale ranging from 0 (“I was not really exposed”) to 10 (“I was one of the people most exposed”).

Peri-traumatic reactions were measured by the Shortness of breath, Tremulousness, Racing heart and Sweating scale (STRS), which is a 13-item scale (ranging to 0 to 4) that provides a retrospective score of the somatic manifestations of fear [24], and the Peritraumatic Dissociative Experience Questionnaire (PDEQ), which is a 10-item (ranging from 0 to 4) questionnaire that measures peri-traumatic dissociative symptoms [25].

#### Psychological support

All participants were interviewed about the psychological support they received (by a professional or a volunteer) in the 48 h after the events, between 48 h and 1 week after, and more than 1 week after. In both waves, participants were also asked if they had had regular care, support or follow-up with a psychologist or psychiatrist after the events. In both waves, they were also interviewed about their overall satisfaction with the psychological support they received from professionals or volunteers, to cope with stress since the events.

#### Perceived social support

In both waves, at the time of the interview(s), all participants were asked about their current perceived feeling of isolation and about current emotional, financial and material support.

#### Impact on work, social and family life

The Sheehan Disability Scale was used to assess functional impairment in three domains: work/school, social, and family life. Each domain score was divided into 5 categories: 0 (unimpaired), 1–3 (lightly impaired), 4–6 (moderately impaired), 7–9 (notably impaired) and 10 (severely impaired) [26]. We recoded each score to a binary variable “declared a score greater than 4 or not”.

#### Experience with media

In Wave 1, participants were asked if they had been contacted and/or interviewed by any media about their experiences with the January 2015 terrorist attacks.

#### Mental health disorders

For both waves, modules from the Mini-International Neuro-psychiatric Interview (MINI) v6 questionnaire were used to assess current PTSD, depression, and anxiety disorders (agoraphobia, social phobia, panic disorder or general anxiety) [27]. In addition, in both waves, interviewers used the Clinical Global Impression – Severity Scale (CGI - S) to rate the current severity of respondents’ general mental health state relative to their past clinical experience with similarly diagnosed patients. This scale has 7 possible ratings (normal, not at all ill, borderline mentally ill, mildly ill, moderately ill, markedly ill, severely ill, and among the most extremely ill patient) [28].

### Statistical analyses

We performed a logistic regression analysis to compare participants lost to follow-up with those who participated in both waves (the dependent variable was “lost to follow-up” (yes/no)). All variables associated with the outcome with a *p*-value < 0.10 were included in an initial multivariate model and then manually backward-selected. Because of the small number of new participants in Wave 2, we could not perform multivariate regression to compare them with people who had participated in both waves. Comparisons between participants in both waves and new participants in Wave 2 were performed using Chi-square tests (or exact Fisher tests when frequencies were low) for the categorical variables and using Mann-Whitney’s tests for the continuous variables (due to the non-normal distribution of the continuous variables), with a two-sided statistical significance level of 0.05. All analyses were carried out using R (version 3.5.2) statistical software.

The dataset supporting the conclusions of this article is included within its additional file.

## Results

### Non-participation in wave 1

**Fig. 1:**
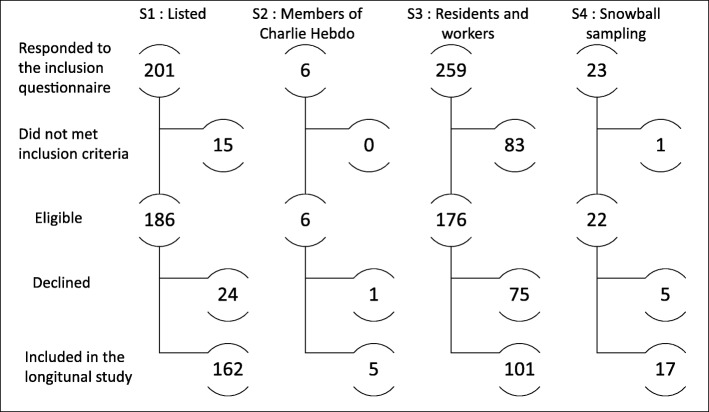
Process for inclusion of participants in the IMPACTS survey according to the different data sources used in the survey

1. Authorities and caregivers listed 410 people as being injured, hostages or witnesses who had to flee the scene because their lives were threatened. In Wave 1, the survey team attempted to contact the 249 persons in this list with an available telephone number, in order to solicit their participation in IMPACTS. Of these, 48 were unreachable. The other 201 were all contacted by a member of the survey team (specifically a psychologist or health professional). Fifteen did not meet the inclusion criteria (Fig. 1). Among the 186 who did, 24 declined to participate (13%) and 162 agreed (agreement rate: 87%).
2. Of the 15 members of the editorial staff of *Charlie Hebdo,* 6 responded to the inclusion questionnaire by e-mail. Five of these members agreed to participate in IMPACTS and 1 person declined (agreement rate: 83%).
3. Of the 884 residents and workers believed to be present within a 100-m radius of the attacks when they occurred, 259 (29%) responded to the invitation letters. Of these, 83 (32%) were excluded because they did not meet the inclusion criteria. Of the 176 who were eligible, 75 (43%) declined to participate in IMPACTS, while 101 agreed (agreement rate: 57%).
4. Twenty-three people were identified as close relatives of people involved using snowball sampling. Of these 1 did not meet the inclusion criteria. Five of the remaining 22 (23%) declined to participate in IMPACTS while 17 agreed (agreement rate: 77%).

To summarize, in Wave 1, of the 489 people who responded to the inclusion questionnaire, 99 did not meet the inclusion criteria (20%). Of the remaining eligible 390, 105 declined to participate (27%) while 285 agreed they would participate in the longitudinal study (global agreement rate to participate in IMPACTS: 73%).

**Fig. 2:**
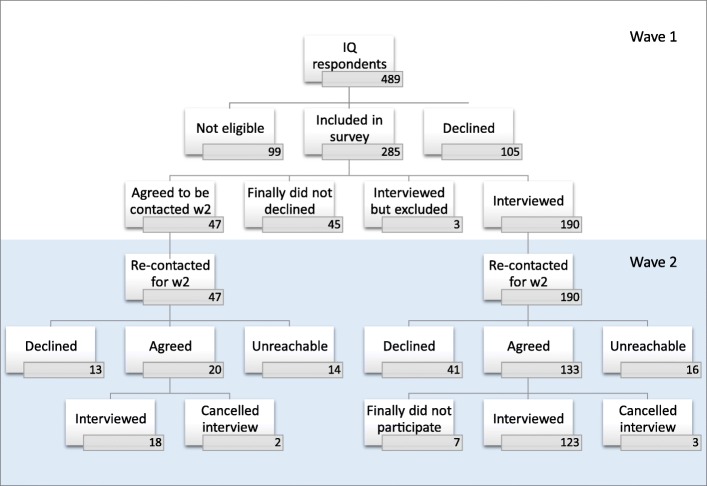
Flow chart of IMPACTS survey (waves 1 and 2)

However, of the 285 who initially agreed to participate, 190 participated in Wave 1, 47 only participated in Wave 2, 45 did not in the end participate in either wave, and 3 were secondarily excluded because of inaccurate reporting (Fig. 2). The actual participation rate in Wave 1 was therefore 49% (190/390).

Among the 105 persons who declined to participate, the reasons cited were a desire not to be reminded of the painful event (32%, *n* = 34), lack of time (16%, *n* = 17), not feeling concerned (15%, *n* = 16) and the fear of data leakage (5%, *n* = 5). Thirty-three people (31%) did not provide any reason for their refusal. Participants were younger than people who declined (39 y/o, 52 y/o, respectively, *p* < 0.001). The gender ratio did not differ between participants and those who declined (1.53 vs 1.30 respectively, *p* = 0.527).

### Attrition in wave 2

#### Participation rate

For the second wave, of the 190 participants in Wave 1, 123 (65%) were re-interviewed, 16 (8%) were unreachable, 41 (22%) declined to participate and 10 (5%) were not interviewed (reasons unknown) or they cancelled the interview. Among those who declined, 17 (42%) did not provide any reason, 7 (17%) reported not having enough time, 5 (12%) preferred to forget the events and to move on without being reminded of them again, 4 (10%) said it was still too painful to talk about what had happened, 3 (7%) said that their participation was useless, 1 (2%) moved away from Paris, and 4 (10%) gave other reasons.

#### Factors associated with attrition in wave 2

In univariate analysis, attrition was significantly higher in younger people (OR_[31–50]_ = 0.41 _95%_CI[0.20–0.84], OR_[≥51]_ = 0.27 _95%_CI[0.11–0.65], *p* = 0.007), in those who had non-French origin (OR = 3.2 _95%_CI[1.01–10.21], *p* = 0.049) (Table 1), those who were impacted by the attacks in the suburban towns of Paris (as opposed to the Paris city centre attack at Charlie Hebdo) (OR = 2.13 _95%_CI[1.13–4.01], *p* = 0.018). Attrition was higher in those who had no support after i) 48 h, ii) at 1 week iii) and/or more than 1 week following the events (OR = 2.00 _95%_CI[1.01–3.93], *p* = 0.046). Attrition was lower in those who lived alone (OR = 0.42 _95%_CI[0.20–0.92], *p* = 0.030) and in those who reported that the events had affected their work moderately or severely(OR = 0.34 _95%_CI[0.18–0.65], *p* = 0.001). We did not observe significant associations between attrition and other factors assessed in this study (other sociodemographic characteristics, exposure characteristics, medico-psychological support, consumption of tobacco, alcohol or cannabis, social support or mental health). As indicated in the Methods, all variables associated with the outcome with a *p*-value < 0.10 were included in an initial multivariate analysis. Thus, we also included regular psychological support after the attacks (*p* = 0.094) and being interviewed by media about their experiences with the attacks (*p* = 0.063).

**Table 1:** Comparison of participants in both waves to those who participated only in Wave 1

	Total *N* = 190		Lost to follow-up *N* = 67		Waves 1 & 2 *N* = 123		OR	CI	*p*-value
	*N/m*	*%/sd*	*N/m*	*%/sd*	*N/m*	*%/sd*			
Socio-demographics									
Female Gender	115	60.5	40	59.7	75	61.0	0.95	[0.85–1.74]	0.864
Age at time of terrorist attack:									
*mean*	41.8	13.6	38.8	14.3	43.5	12.9			
*min-max*	19–84		19–84		23–79				
[18–30]	49	25.8	26	38.8	23	18.7	ref		**0.007**
[31–50]	94	49.5	30	44.8	64	52.0	0.41	[0.20–0.84]	
≥ 51	47	24.7	11	16.4	36	29.2	0.27	[0.11–0.65]	
Educational Level < high-school diploma	57	30.2	24	35.8	33	27.0	0.66	[0.35–1.26]	0.210
Unemployed	34	17.9	14	20.9	20	16.3	1.36	[0.64–2.91]	0.427
Living alone	46	24.2	10	14.9	36	29.3	0.42	[0.20–0.92]	**0.030**
Non-French origin	13	6.8	8	11.9	5	4.1	3.20	[1.01–10.21]	**0.049**
Terror exposure									
Event location outside of Paris (vs Paris)	131	68.9	39	58.2	92	74.8	2.13	[1.13–4.01]	**0.019**
Geographic exposure:									
*Less than 10 m*	20	10.5	6	9.0	14	11.4	1.39	[0.32–6.08]	0.639
*Very close, next room*	64	33.7	23	34.3	41	33.3	1.82	[0.53–6.25]	
*Neighbouring building*	89	46.8	34	50.7	55	44.7	2.01	[0.61–6.67]	
*Elsewhere*	17	8.9	4	6.0	13	10.6	ref		
Objective exposure:									
*Directly threatened*	58	30.5	22	32.8	36	29.3	0.86	[0.37–2.00]	0.516
*Indirectly threatened*	82	43.2	27	40.3	55	44.7	0.38	[0.09–1.61]	
*Close relative of victims*	14	7.4	3	4.5	11	8.9	0.69	[0.31–1.54]	
*Witness*	36	18.9	15	22.4	21	17.1	ref		
Perceived terror exposure (0–10)	6.3	3.0	6.1	3.1	6.4	3.0	0.97	[0.88–1.07]	0.518
Peri-traumatic reactions									
PDEQ (0–40)	15.5	10.5	14.2	10.8	16.2	10.3	0.98	[0.95–1.01]	0.226
STRS (0–52)	22.5	11.6	20.9	12.9	23.4	10.9	0.98	[0.96–1.01]	0.170
Sheehan disability scale									
Work (impact ≥4)	83	45.1	19	28.8	64	54.2	0.34	[0.18–0.65]	**0.001**
Social life (impact ≥4)	75	39.5	22	32.8	53	43.1	0.65	[0.35–1.20]	0.168
Family life, home responsibilities (impact ≥4)	66	34.7	23	34.3	43	35.0	0.97	[0.52–1.82]	0.930
Psychological support									
No psychological support (after 48 h, 1w, or > 1w)	46	24.2	22	32.8	24	19.5	2.00	[1.01–3.93]	**0.046**
No regular psychological follow-up after attack	144	76.2	55	83.3	89	72.4	1.91	[0.89–4.08]	**0.094**
Dissatisfaction with psychological support from professionals	56	32.6	22	39.3	34	29.3	1.56	[0.80–3.05]	0.192
Media impact									
Contacted by media regarding the event	116	61.1	40	59.7	76	61.8	0.92	[0.50–1.68]	0.778
Interviewed by media regarding the event	63	34.8	28	43.8	35	29.9	1.82	[0.97–3.43]	**0.063**
Social support									
Feeling alone	27	14.2	7	10.4	20	16.3	0.60	[0.24–1.50]	0.277
No perceived moral/emotional support	2	1.1	1	1.5	1	0.8	1.85	[0.11–30.03]	0.666
No perceived financial/material support	37	19.5	11	16.4	26	21.1	0.73	[0.34–1.60]	0.434
No perceived everyday support	20	10.5	6	9.0	14	11.4	0.77	[0.28–2.10]	0.603
Mental health									
PTSD (previous month)	34	17.9	14	20.9	20	16.3	1.36	[0.64–2.91]	0.427
Major depressive disorder (last two weeks)	20	10.5	7	10.4	13	10.6	0.99	[0.37–2.61]	0.979
Suicide risk (previous month)	52	27.4	15	22.4	37	30.1	0.67	[0.34–1.34]	0.257
Anxiety disorders (at least one)	50	26.5	16	23.9	34	27.9	0.91	[0.47–1.73]	0.764
Moderately/markedly/severely ill (CGI)	55	29.4	17	26.2	38	31.1	0.78	[0.40–1.53]	0.476

IMPACTS survey, France, 2015

*N* Number, *m* Mean, *sd* Standard deviation, *OR* Odds ratio, *CI* Confidence interval, *ref* Reference. *P*-value was two-sided statistical significance level of the logistic regression. *P*-values in bold were lesser than 0.10

Apart from psychological support after the event and interviews by different media, all study variables were significantly associated with attrition in Wave 2 in multivariate analysis (Table 2). People at least 31 years old participated in both waves significantly more often than younger participants (OR_[31–50]_ = 0.34 _95%_CI[0.15–0.75], OR_[≥51]_ = 0.14 _95%_CI[0.05–0.40], *p* < 0.001). Attrition was higher in participants who had non-French origin (OR = 5.83 _95%_CI[1.51–21.52], *p* = 0.009) and in those who were impacted by the attacks in the suburban towns of Paris (as opposed to the Paris city centre attack at Charlie Hebdo) (OR = 2.19 _95%_CI[1.06–4.53], *p* = 0.033). Attrition was lower in those who lived alone (OR = 0.37 _95%_CI[0.15–0.88], *p* = 0.019) and those who had a Sheehan work score greater than 4 (OR = 0.47 _95%_CI[0.23–0.97], *p* = 0.012).

**Table 2:** Multivariate regression analysis of the comparison between those were lost to follow-up and those who participated in both waves

	OR	CI	*p*-value
Age at time of the attack			< 0.001
[18–30]	Ref.		
[31–50]	0.34	[0.15–0.75]	
≥ 51	0.14	[0.05–0.40]	
Living alone			0.019
No	Ref.		
Yes	0.37	[0.15–0.88]	
French Origin			0.009
French origin	Ref.		
Non-French origin	5.83	[1.51–22.52]	
Event location			0.033
Paris	Ref.		
Suburban town	2.19	[1.06–4.53]	
Sheehan Work score			0.012
< 4	Ref.		
≥ 4	0.41	[0.21–0.83]	

IMPACTS survey, France, 2015.

*OR* Odds ratio, *CI* Confidence interval, *Ref* Reference. *P*-value was the two-sided statistical significance level of the logistic regression.

### New study participation: wave 2

Among the 47 eligible civilians who agreed to participate in Wave 2 but not Wave 1, 18 did in fact participate (38%), 15 (32%) were unreachable and 14 (30%) declined when re-contacted for Wave 2 (Fig. 2). The reasons cited by the latter were: “it is still too painful” (*n* = 3), “I prefer to forget these tragic events and to move on without being reminded again” (*n* = 3), “my participation is useless” (*n* = 2), “I do not have enough time” (*n* = 2). Four did not provide any reason for their non-participation.

The 18 new study participants were significantly more likely to report no regular psychological support or follow-up than those who participated in both waves (72.2% vs 38.2%, *p* = 0.010) (Table 3). They were also more likely to feel alone in 2015 after the attacks (44.4% vs 16.3%, *p* = 0.005) and to report having no moral support when needed (22.2% vs 0.8%, *p* = 0.001). This difference in the level of social support provided was not observed in Wave 2.

**Table 3:** Comparison of participants who participated in both waves with new participants in Wave 2

	Total wave 2 *N* = 141		New participants *N* = 18		Wave 1 & 2 N = 123		*p*-value*
	N/m	%/sd	N/m	%/sd	N/m	%/sd	
Socio-demographics							
Female Gender	82	58.2	8	44.4	75	61.0	0.183
Age at time of the attack:							
*mean, sd*	42.9	12.9	39.1	12.0	43.5	12.9	0.205
*min-max*	21–79		21–60		23–79		
Educational Level < high-school diploma	41	29.3	8	44.4	33	27.0	0.130
Unemployed at Wave 2	37	26.2	5	27.8	32	26.0	0.999
Living alone at Wave 2	46	32.6	7	38.9	39	31.7	0.594
Non-French origin	8	5.7	3	16.7	5	4.1	0.065
Terror exposure							
Event location outside of Paris (vs Paris)	39	27.7	8	44.4	31	25.2	0.088
Geographic exposure:							
*Less than 10 m*	20	14.2	6	33.3	14	11.4	NA
*Very close, next room*	46	32.6	5	27.8	41	33.3	
*Neighbouring building*	62	44.0	7	38.9	55	44.7	
*Elsewhere*	13	9.2	0	0	13	10.6	
Objective exposure:							
*Directly threatened*	44	31.2	8	44.4	36	29.3	NA
*Indirectly threatened*	60	42.6	5	27.8	55	44.7	
*Close relative of victims*	11	7.8	0	0	11	8.9	
*Witness*	26	18.4	5	27.8	21	17.1	
Perceived terror exposure (mean 0–10)	6.3	2.9	5.9	2.7	6.4	3.0	0.378
Sheehan disability scale in Wave 2							
Work (impact ≥4)	69	48.9	7	38.9	62	50.4	0.452
Social life (impact ≥4)	67	47.5	8	44.4	59	48.0	0.806
Family life, home responsibilities (impact ≥4)	53	37.6	6	33.3	47	38.2	0.798
Medical/psychological support							
No regular psychological support or follow-up since the event	60	42.6	13	72.2	47	38.2	**0.010**
Dissatisfaction with psychological support from professionals since the events	56	40.0	8	47.1	48	39.0	0.526
Social support							
Feeling alone in 2015	28	19.9	8	44.4	20	16.3	**0.005**
Feeling alone in 2016	34	24.1	7	38.9	27	22.0	0.117
No perceived moral/emotional support in 2015	5	3.5	4	22.2	1	0.8	**0.001**
No perceived moral/emotional support in 2016	6	4.3	2	11.1	4	3.3	0.169
No perceived financial/material support in 2015	30	21.3	4	22.2	26	21.1	0.999
No perceived financial/material support in 2016	28	19.9	4	22.2	24	19.5	0.757
No perceived support in everyday life 2015	18	12.8	4	22.2	14	11.4	0.249
No perceived support in everyday life 2016	17	12.1	3	16.7	14	11.4	0.457
Physical and psychological health							
Deterioration of general health state	27	19.1	3	16.7	24	19.5	0.999
Deterioration of psychological health state	28	19.9	2	11.1	26	21.1	0.527
Having at least one health problem	135	95.7	17	94.4	118	95.9	0.627
Mental health at Wave 2							
PTSD (previous month)	21	15.0	3	17.6	18	14.6	0.721
Major depressive disorder (last two weeks)	12	8.6	2	11.8	10	8.1	0.641
Suicide risk (previous month)	57	40.7	4	23.5	53	43.1	0.187
Anxiety disorders (at least one)	54	38.6	7	41.2	47	38.2	0.814
Moderately/markedly/severely ill (CGI)	42	31.8	3	33.3	39	31.7	0.839
Involvement in November 2015 attacks							
Directly threatened or witness	9	6.4	0	0	9	7.3	NA
Close relative of victims	16	11.3	2	11.1	14	11.4	0.999

IMPACTS survey, France, 2015–2016

*N* Number, *m* Mean, *sd* Standard deviation, *NA* Non available. *P*-value was the two-sided statistical significance level of the chi-square test for categorical variables and Mann-Whitney test for continuous variables. *P*-values in bold were lesser than 0.05)

No significant differences were observed between these new participants and those who participated in both waves in terms of other sociodemographic characteristics, exposure characteristics, medico-psychological support, toxic substance consumption, social support or mental health.

## Discussion

Twenty years after the first study on terrorist attacks in France [29], the IMPACTS survey investigated the impact of the Paris January 2015 terrorist attacks on the mental health and social functioning of civilians involved. The present study enabled us to describe non-participation in Wave 1 of the survey and determine the factors associated with attrition in Wave 2. More specifically, this study focused on the number of persons solicited to participate in IMPACTS, on those who initially agreed/declined to participate, and on those who actually participated. Potential participants for IMPACTS came from a variety of sources, with different stakeholder profiles: survivors, residents, workers, witnesses, as well as close relatives of those injured, those taken hostage and those who died. Given that the total number of civilian stakeholders in these terrorist attacks is unknown, it was not possible to compute global participation rates. Among all the people contacted who met the survey’s inclusion criteria (*N* = 390), the final participation rate for Wave 1 was 49%. The participation rate for these same people was 65% in Wave 2.

In the last 20 years, cohort studies dealing with the health impacts of terrorist attacks on civilians have been conducted in the US (following the 11 September, 2001 terrorist attacks), and in Europe (following the 11 March, 2004 train bombings in Madrid, and the Oslo/Utøya massacre in 2011 [30]). Participations rates in these cohorts ranged from 40 to 70% in the first study waves, and were generally higher in subsequent waves (50 to 75%).

Comparing the participation rates of these studies is unrealistic because of the differing study contexts, designs and populations. If a terrorist attack occurs in a public place and involves the general population, those who are directly threatened may be more heterogeneous than an attack which occurs in specific groups or communities, such as the Utøya island attack where the perpetrator attacked a summer camp hosting members from the Norwegian Labour Party’s youth organization [31]. This heterogeneity suggests the need for tailored strategies to recruit representative participants and involves different designs. Indeed, cohorts on this theme have been recruited from Web-enabled panels [32, 33], registries [34], authority lists, healthcare centres [35], and random digit dial telephone surveys [36, 37]. Moreover, some studies have involved only those directly threatened whereas others, like IMPACTS, also involved witnesses, close relatives, residents and workers present during the attacks. For example, the World Trade Center Health Registry (WTCHR) cohort comprised not only people present at the 9/11 attack site itself (workers and volunteers involved in rescue, recovery, clean-up, and other activities) but also residents in Canal Street in lower Manhattan, and students and staff employed at schools south of Canal Street [34]. In addition, the numbers of participants differ greatly between cohorts, ranging from a few hundred to many thousands of participants. For example, the WTCHR cohort enrolled more than 71,000 people exposed to the 9/11 attack [38] while 1589 individuals participated in a longitudinal study after the Madrid train attack [37], and 355 were enrolled in the open cohort of Utøya [21]. The moment when the investigation takes place and the length of time between successive study waves, may influence participation, and is very heterogeneous between the cohorts mentioned here. For instance, two of the cohort studies above were based on a nationally representative, web-enabled panel which was collected 10 days after the 9/11 attacks [32, 33] while the Utøya cohort interviewed participants for the first time 4 to 5 months after the events [21].

In France, only three studies on terror attacks were conducted in the past, following the 20 bombing and 1 machine-gun attacks that occurred in the country between 1982 and 1987 [29], and another wave of bombing attacks that occurred in 1995 and 1996 [39, 40]. *Abenhaim* et al. conducted a retrospective cross-sectional survey on 254 survivors listed by the police and medical emergency services after the 1982–1987 terrorist attacks [29]. The participation rate in that study was 78% (*n* = 254/324). In *Verger* et al.’s retrospective cross-sectional study on the 1995–1996 terrorist attacks among people identified as “victims” by the French Terrorism Victim Guarantee Fund [39], the participation rate was 86% (*n* = 196/228). Finally, *Jehel* et al. published a study on the 1995–1996 terrorist attacks among civilians listed by the police department and by an organization that provided psychological and juridical support to the victims [40]. Among the 111 people contacted 6 months after the events, 51% participated in the first wave, of whom 55% participated in the second wave one year after.

Attrition in Wave 2 was higher in participants aged 18 to 30 y/o than in older participants, and in people of non-French origin. The latter finding was also observed in the Utøya study for people of non-Norwegian origin [21] and a cohort study of the survivors from the World Trade Center terrorist attack [20]. The age effect has also been observed in health surveys in the French general population [15] and in trauma-related studies [18, 20, 21]. Both results suggest that it is important to develop innovative recruitment strategies for younger participants and for people of non-French origin for future waves of the present study as well as for future studies in the context of other terror attacks. Attrition was higher for participants living in suburban towns, perhaps because of relatively higher recruitment in Paris. Participants exposed to the city-centre attack (Charlie Hebdo) may have had better healthcare management in the wake of the attack because of greater available services in the city of Paris than in its suburbs. Moreover, they may have been more willing to talk to the survey’s investigating psychologists and to participate [41]. Indeed, in univariate analysis, those lost-to follow-up were more likely not to have had psychological follow-up after the events. Since attrition did not seem to be a consequence of either the terror exposure level or mental health problems, we hypothesize that the risk of under- or over-estimation of the prevalence of mental health disorders due to the consequences of the attacks in Wave 2 was limited.

Although impacted by the small numbers of individuals involved, the comparison between the profiles of new participants in Wave 2 and people who participated in both waves highlights the value of conducting an open cohort study to be able to include specific victim profiles at a later date. We found that new participants were more socially isolated: they were more likely to feel alone and to report no moral or emotional support at Wave 1 (but less likely at Wave 2), which strongly suggests that their initial social isolation might have negatively influenced their decision to participate initially. This result was consistent with the study of Stene et al. that indicated that non-participation was associated with less social support [21]. Furthermore, new participants were more likely not to have had any regular psychological support after the attacks. Finally, although the small sample size prevented the possibility of performing a significance test, new participants were more likely to have been directly threatened or geographically very close to the terrorist(s), i.e. they were more likely to be those most exposed.

The IMPACTS survey had strengths and limitations in terms of recruitment. The first limitation is that the initial questionnaire was in French only and this may have excluded potential participants who did not speak French. Second, the investigation period for both waves (between June and October) may not have been the most suitable choice as it covered the summer holidays, when many people go on vacation. Furthermore, during the second study wave, another terror attack occurred in the city of Nice, during the traditional Bastille Day fireworks festival (14 July, 2016) where 500 people were injured and 86 were killed [42]. Even though most, if not all of the people exposed to the Paris attacks were not physically in Nice when this attack occurred, they were very probably exposed to images and testimonies widely publicised on the media. It is well known that re-exposure can reactivate PSTD symptoms [43, 44]. Accordingly, the Nice attack may have influenced participation in Wave 2 of IMPACTS.

The strengths of the IMPACTS survey in terms of recruitment were, first, the combination of several lists from different authorities, the comprehensive research carried out to estimate the number of residents and workers who were potentially present when the events took place, and the pro-active search for people which were not listed (mainly witnesses and close relatives). Second, the IMPACTS survey focused a great deal on ethical considerations, something which may have maximized participation. In particular, all the interviewers were psychologists trained in trauma. They worked at avoiding re-activation of PSTD symptoms and referred participants to selected mental health services if needed. A hotline was created to facilitate the sharing of information to and between collaborating psychologists and psychiatrists, and to help orient participants with specific psychological needs. Furthermore, as much as possible, participants were interviewed during both waves by the same psychologist. For participants who had moved residence between the two waves, the interviewer proposed meeting them at their new home or doing the interview by video.

### Study limitations

The present study has other limitations. First, the total number of civilians exposed was unknown because the total numbers of residents and workers present at the time of the attacks was not available. Furthermore, the total number of people close to the injured, to those taken hostage and to those who died was also unavailable. In the absence of such a denominator, the “true” participation rate could not be estimated. Nor was it possible to estimate bias due to the non-participation of unidentified, uninformed people who met the inclusion criteria (“to be involved in the terrorist attacks of January 2015 according to the exposure criteria A for Posttraumatic Stress Disorder (PTSD) of the DSM-5”). However, we can reasonably suppose that all those directly exposed were identified and listed by the authorities.

Second, the number of new participants in the second wave was small. Consequently, we were not able to generate reliable estimates and robust comparisons with those who participated only in Wave 2. Finally, only declarative data were available and we cannot exclude the possibility that some of these data were subject to recall or reporting bias.

## Conclusions

The lower level of participation of younger people (i.e., < 31 y/o), of people of non-French origin, and In those who were impacted by the attacks in the suburban towns of Paris (as opposed to the Paris city centre attack at Charlie Hebdo) suggests that other recruitment strategies are necessary to improve participation by these subpopulations in future waves of the IMPACTS study and in future surveys following terrorist attacks in general. Indeed, following the IMPACTS study, some recommendations have already been made for strategies to encourage such participation. The first is to display information posters in town council halls, local social service structures, and community-based organizations’ premises (for the young, the elderly, migrants, minorities, etc.) in areas exposed to terror, as well as in Medico-Psychological Emergency Units (called CUMP in France), and common consultation sites. The second is to involve social media [45, 46]. For younger people in particular, this could be achieved by mobilizing educational institutions and professionals. A third possible strategy to encourage participation, is the implementation of community-based collaborative research processes which have been proven to increase trust between research partners and participants, and increase engagement of hard-to-reach populations [47]. In 2013, a symposium suggested integrating research and evaluation into disaster-response planning [48].

In the absence of existing guidelines on health research after mass trauma, we would recommend that future guidelines need to ensure that great attention is paid to the ethical and methodological issues and challenges involved in any study investigating the consequences of mass trauma on mental health and social functioning. Notably, we suggest i) to make efforts to collect information of non-participants in an ethically respectful way, ii) to use an open cohort design in longitudinal post-terror studies in order to allow for later participation among survivors who are unable to participate in the first wave during the early aftermath of the attack, and iii) to systematically estimate levels of and factors associated with non-participation and attrition to take into account potential selection bias in the interpretation of the findings.

## Data Availability

The data that support the findings of this study are available from The French Public Health Agency (*Santé Publique France*), but restrictions apply to the availability of these data, which were used under license for the current study, and so are not publicly available. Data are however available from the authors upon reasonable request and with permission of The French Public Health Agency.

## Abbreviations

CI: Confidence Interval
CUMP: French acronym for medical-psychological emergency unit (*Cellule d’Urgence Médico-Psychologique* in French)
DSM-5: Diagnostic and Statistical Manual of Mental Disorders, fifth edition
IMPACTS: French acronym for Investigation of Trauma Consequences in People Exposed to the January 2015 Terrorist Attacks and their Support and Mental care (*Investigation des manifestations traumatiques post-attentats et de la prise en charge thérapeutique et de soutien des personnes impliquées dans les attentats de janvier 2015 en Île-de-France* in French)
MINI: Mini-International Neuro-psychiatric Interview
OR: Odds ratio
PDEQ: Peritraumatic Dissociative Experience Questionnaire
PTSD: Posttraumatic Stress Disorder
STRS: Shortness of breath, Tremulousness, Racing heart and Sweating scale
WTCHR: World Trade Center Health Registry
